# Experiment Study on the Effect of Aluminum Sulfate-Based Alkali-Free Accelerator and the *w*/*c* on Cement Hydration and Leaching

**DOI:** 10.3390/ma16062165

**Published:** 2023-03-08

**Authors:** Xuepeng Ling, Quande Wu, Jie Yang, Wenzheng Wang, Dagang Liu, Yiteng Zhang

**Affiliations:** 1 Key Laboratory of Transportation Tunnel Engineering of Ministry of Education, Southwest Jiaotong University, Chengdu 610031, China; 2 School of Civil Engineering, Southwest Jiaotong University, Chengdu 610031, China; 3 China Railway Economic and Planning Research Institute Co., Ltd., Beijing 100038, China

**Keywords:** aluminum sulfate-based alkali-free accelerator, Ca-leaching, hydration kinetics, accelerated leaching test, pore structure

## Abstract

The alkali-free accelerator based on aluminum sulfate is widely used in shotcrete in tunnels. Long-term Ca-leaching of shotcrete may adversely affect the tunnels in a water-rich mountain. It is necessary to examine further the impact of the AS accelerator and *w*/*c* on cement hydration and leaching. In this study, all the cement pastes were cured in the environment with R.H. > 95% and 20 ± 1 °C for 60 days and leached in a running water test with 6 M NH_4_Cl at 1 cm/s. The hydration kinetics was characterized by isothermal calorimetry. Additionally, the microstructural and mineralogical alterations were characterized by XRD, SEM, MIP, and N_2_ absorption. The results show that (1) the AS accelerator affected the hydration kinetics of cement by stimulating early hydration and delaying the late silicate hydration, resulting in AS-accelerated cement pastes with rougher pore structure. As a result, the higher the dose of AS accelerator, the faster the cement pastes will leach. (2) Hydration kinetics of the accelerated cement are not affected by the *w*/*c*. The AS-accelerated cement pastes with lower *w*/*c* have a denser pore structure. So, the reduction in the *w*/*c* contributes to leaching resistance.

## 1. Introduction

**Figure 1:**
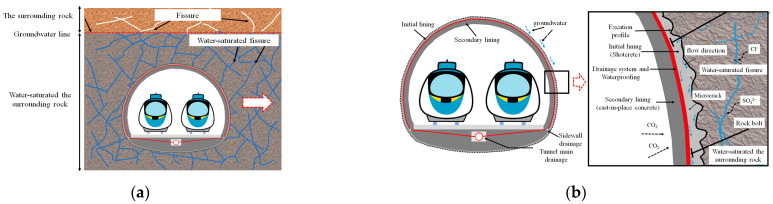
The composite lining structure of the drill-and-blast tunnel in the water-rich mountain: (**a**) water-rich mountain tunnel; (**b**) a composite lining structure of the tunnel.

Drill-and-blast tunnels excavated in China usually employ a composite lining structure, which includes an initial lining and a secondary lining, as shown in Figure 1. The initial lining, typically, is shotcrete or section steel–shotcrete [1], and the secondary lining is molded concrete or reinforced concrete. The two linings share the load generated by the deformation of the loose surrounding rock. Between the initial and secondary lining, drainage systems and waterproof layers are usually installed to reduce groundwater’s interference with traffic. The secondary lining is protected from the intrusion of groundwater, whereas the initial lining is not. Shotcrete faces serious durability issues when exposed to groundwater environments such as soft water or sulfate for long periods, putting the long-term safety of the tunnel structure at risk.

A soft water environment, causing shotcrete to leach calcium more quickly, is common in water-rich tunnels in mountainous terrain. Leaching is defined as a process of dissolving cement hydration products [2,3] and diffusing calcium ions into the surrounding environment [4,5]. In leached concrete, the pores increase greatly, and the density decreases, resulting in a reduction in strength [6,7].

**Figure 2:**
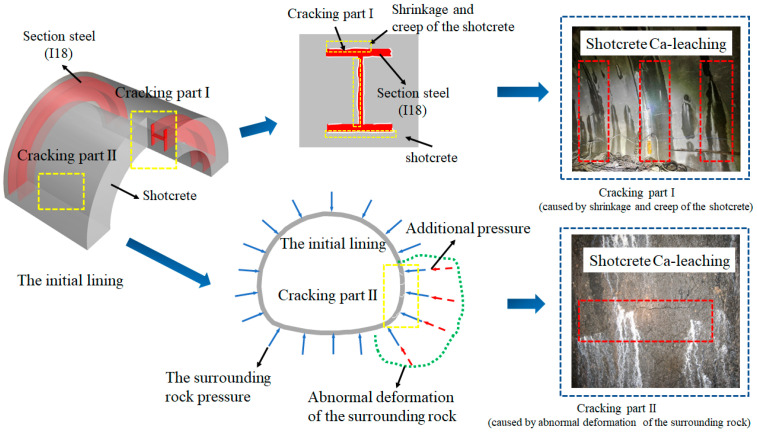
Survey of shotcrete leaching in the field.

Through investigation of several water-rich mountain tunnels in southwestern China, it was found that groundwater leaks occurred either at the intersection of the profile steel shotcrete or at the site where the shotcrete was cracked and that such leaked concrete had signs of relatively significant leaching (to be shown in Figure 2). The former is caused by shrinkage and creep of the shotcrete, while the latter is caused by abnormal deformation of the surrounding rock [8], which results in the cracking of the shotcrete. The natural leaching of concrete is a prolonged process [9,10]. Although its impact is insignificant in initial lining during the completion phase of the tunnel for a limited time, the process cannot be ignored for civil engineering projects with a service life of up to 100 years, such as traffic tunnels. It weakens material properties, reduces bearing capacity, and thus threatens the structural integrity of the tunnel [11,12]. 

Due to the time-consuming leaching [13,14] under natural conditions [15], the current study of concrete leaching mainly adopts accelerated test [16,17,18], of which the most common is the chemical accelerated leaching method represented by NH_4_NO_3_/NH_4_Cl solution [19]. Experimentally, Wan et al. determined that the chemical acceleration leaching process is similar to that of natural conditions [20,21]. Reducing the *w*/*c* and substituting clinker with supplementary cementitious materials (SCMs) enhanced the pore structure of cement pastes, which will improve their leaching resistance. As a result of the accelerated leaching method of NH_4_NO_3_ solution used by Wan et al. [20], they concluded that cement pastes with low *w*/*c* are more resistant to leaching. Zuo et al. proposed that the SCMs, such as blast furnace slag, increase their leaching resistance [22] based on an NH_4_Cl solution. In the above studies, the main research methods and factors influencing the Ca-leaching process in cement without AS accelerator/ordinary molded concrete have been summarized. Nevertheless, the research objects primarily involve the AS accelerator cement or shotcrete, so it remains to be determined whether the findings of these studies can be applied.

Shotcrete [23] is a type of concrete in which aggregates, water, and binders are mixed in advance, pumped into a nozzle together with an accelerator and compressed air, and sprayed onto the substrate. Then, with the accelerator, the concrete sprayed into the substrate can harden within minutes. Such concrete can be placed without additional support, and increase in strength rapidly in the early stages. Accelerators are generally classified into two groups based on their chemical composition: alkaline and alkali-free accelerators. In the alkaline accelerator based on sodium aluminate solution [24], the gypsum or anhydrite of the cement is consumed quickly at the beginning and, at the same time, ettringite and AFm phases are produced to accelerate the cement hardening process. The massive ettringite and AFm phases [25,26,27] formation at an early age fill up the matrix, and less space remains for the alite hydration [28,29] to proceed. Due to the alkali-free accelerator based on aluminum sulfate solution (the AS accelerator) containing a small amount of sulfate, the early hydration reaction produces mainly ettringite and fewer AFm phases, which plays a significant role in cement hardening. As the AS accelerator has less impact on alite hydration than alkaline aluminate accelerators, sprayed concrete mixed with the AS accelerator has a higher early strength [30]. Additionally, the alkaline accelerator may have a negative effect on the health of construction workers. In highway and high-speed railway tunnels in China, the AS accelerator is widely used. The property of the AS accelerator [31], such as Al^3+^ content and Al_2_O_3_/SO_4_^2-^ molar ratio, etc., and accelerator dosage affect the early hydration process of cement, which alters its microstructure and therefore affects its mechanical properties [32,33,34]. The above research has demonstrated that cement mixed with the AS accelerator is different from ordinary cement. However, relevant research is primarily focused on the early stage of cement hydration and rarely looks at the later stage of hydration, which cannot reveal the full impact of the AS accelerator on cement hydration. Furthermore, little research has been conducted on how *w*/*c* affects the hydration of the AS accelerator cement.

The initial lining of a tunnel located in a mountain water-rich region can be exposed to a soft water environment for an extended period. The leaching of shotcrete is, therefore, a major concern; however, there are fewer studies on this subject. It is unclear whether the use of the AS accelerator affects the resistance of shotcrete to leaching. A further investigation of the effects of the AS accelerator on cement hydration, both during the early and later stages, is imperative to assess the leaching resistance of the cement. Furthermore, reducing *w*/*c* is a common method to improve cement’s leaching resistance; however, less research has been conducted on the inclusion of the AS accelerator cement.

In this paper, experimental research is conducted on the influence of the AS accelerator and *w*/*c* cement hydration and leaching. All the cement pastes were cured in the environment with R.H. > 95% and 20 ± 1 ℃ for 60 days. Given that shotcrete leaching in the field investigation occurred in a slow-flowing water environment, this study used the accelerated leaching test with running water with 6M NH_4_Cl at 1 cm/s. The leaching resistance of cement with and without the AS accelerator by determining the leaching depth at various times. Details regarding the microstructural alterations and mineralogy in cement at different hydration times and leaching times were determined by isothermal calorimetry, XRD, SEM, MIP, and N_2_ absorption.

## 2. Materials and Methods

### 2.1. Material

In this study, all cement pastes used Chinese standard ordinary Portland cement (P.O 42.5). The chemical composition and the mineralogical phase composition of Portland cement are shown in Table 1 and Table 2, respectively.

This paper studied the hydration of cement in shotcrete with alkali-free accelerators. The alkali-free accelerator is mainly composed of Al_2_(SO_4_)_3_. The main components and basic properties of the accelerator (based on the Chinese national standard GB/T 35159) [35] are shown in Table 3.

All the cement paste compositions used in this experimental program are shown in Table 4. In general, the cement pastes with different doses of the AS accelerator were categorized into 0% (PA0-045), 4% (PA4-045), and 9% (PA9-045), all with a fixed *w*/*c* equal to 0.45. Additionally, the cement pastes with different *w*/*c* were categorized into 0.40 (PA9-040), 0.45 (PA9-045), and 0.50 (PA9-050), all with a fixed dose of the AS accelerator equal to 9%. All the cement paste samples used 0.8% of superplasticizer by cement weight.

For the preparation of cement pastes, cement, deionized water, and the superplasticizer were mixed by a planetary mixer for 60 s. After the 10 min rest period, the accelerator was added employing a pipette while mixing for 30 s. The fresh cement paste was cast in the cylindrical mold (Φ50 mm × H100 mm) and cured for 24 h at room temperature. After that, these samples were de-molded and continuously cured for 60 days in the curing box at an environmental temperature of 20 °C (±1 °C) with a relative humidity greater than 95%.

### 2.2. Methods

#### 2.2.1. The RNW Test

**Figure 3:**
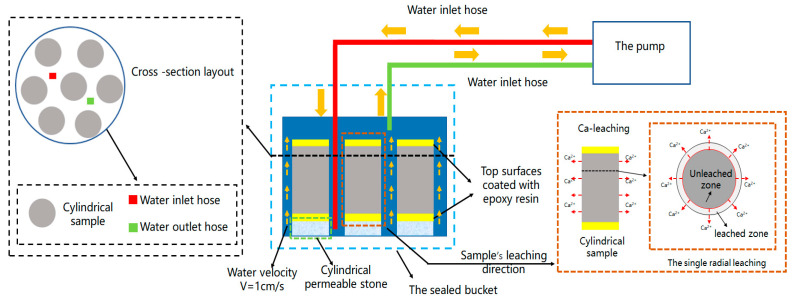
Schematic of the RNW test.

Given that shotcrete Ca-leaching in the field investigation occurred in a slow-flowing water environment, a simulation device (The RWN test device) was designed in this study. The RWN test device consisted mainly of a pump, two hoses, and a sealed bucket (to be shown in Figure 3. Before the test, both end faces of specimens were coated with epoxy resin, while the cylindrical face of the sample served as the calcium leaching surface. Seven similar samples were arranged in a quincunx shape in a sealed bucket with a diameter of about 15.6 cm and then immersed in 6M NH_4_Cl. The running water in the sample cross-section at about 1 cm/s by adjusting the pump. Considering the solid–liquid ratio is greater than 1/800 [36], it was necessary to refresh the NH_4_Cl by the pump every two days during the test period to guarantee an equitable accelerated leaching rate. The accelerated leaching test lasted 35 days. An evaluation of leaching was conducted on three samples of the same type every five days by cutting off an area 1 cm from the bottom of each sample as a test block. After that, the samples were sealed and put back into the device to continue the test.

#### 2.2.2. Test Methods

1. Isothermal calorimetry

The heat flow of all the cement paste samples was recorded with the isothermal calorimeter (TA/TAM-Air-8). The fresh cement paste was transferred into the isothermal calorimeter and left for 36 h at 20 °C.

2. X-ray diffraction

X-ray diffraction (XRD) measures changes in the crystalline phase associated with cement hydration and accelerated leaching. The powder was examined by an X-ray diffractometer (Bruker D8 Advance, Karlsruhe, Germany) with Cu (Kα) radiation and a current of 40 mA and 40 kV. The measurement was conducted at a 4°/min speed within the range from 5 to 70°. Prior to the test, the samples were crushed, and hydration was stopped with solvent exchange by isopropanol for 14 days [37]. The stopped samples were then ground by hand in an agate mortar. The data obtained were analyzed by Rietveld analysis using the software X’Pert High Score Plus. The α-Al_2_O_3_ (10.0% bcw) [38] was added to the powder sample as an internal standard to determine the amorphous content of cement paste. All structure models used for Rietveld refinement were recommended by [24]. Considering the low degree of crystallinity of this material, an indirect method for assessing C–S–H content has been employed by quantifying the amorphous phases [39].

3. Scanning electron microscopy

The SEM test was used to describe qualitatively the microstructure, which was performed in thermo scientific Apreo 2C, with a minimum spot size of 1.2 nm and an accelerating voltage of 10 kV. An SEM morphology observation was performed on cement samples with flat surfaces prepared by solvent exchange [37], as well as EDS energy spectrum analysis of the hydration product elements.

4. Porosity

MIP negatively impacts measurements of smaller pores (<50 nm), since early-hydration samples and leached samples [6,15] developed large deformations at high mercury pressures, whereas N_2_ adsorption performed better. A combination of MIP and N_2_ adsorption was used to quantify the sample of microstructure. Samples with larger pores (>50 nm) were analyzed by MIP experiments with AutoPore IV 9520, while those with smaller pores were analyzed by N_2_ adsorption with V-Sorb 2800P. For a continuous curve, the porosity calculated by N_2_ adsorption was shifted at the combined pore diameter (50 nm) [6]. The samples were prepared by solvent exchange [37].

5. Leaching length

**Figure 4:**
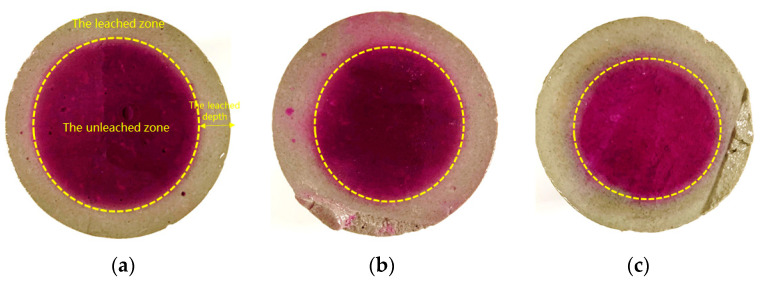
The leaching depth of the cement paste samples in the 35-day accelerated leaching test: (**a**)PA0-045; (**b**) PA9-045; (**c**) PA9-050.

The leaching depth of the sample was measured by the phenolphthalein test [19,20,21]. The fresh surface of leached test block was sprayed with phenolphthalein solution. The phenolphthalein in the leaching area would not turn pink in Figure 4, indicating that calcium hydroxide was leached entirely. The average leaching depth $\bar{h}$ [22] can be calculated by $\bar{h}=R_{o}\left(1-\sqrt{\frac{A_{sound}}{A_{o}}}\right)$, where $\bar{h}$ is the average leaching depth; $R_{o}$ is the radius of the cement sample; $A_{sound}$ is the area of the sound zone which is unleached zone in Figure 4; $A_{sound}$ is the area of the cement sample.

## 3. Results

### 3.1. Isothermal Calorimetry

**Figure 5:**
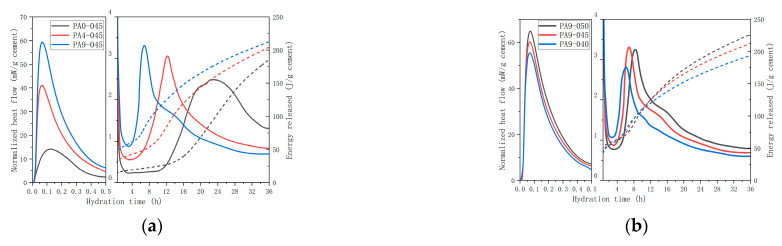
Curves of isothermal calorimetry for AS accelerator cement paste: (**a**) samples with different doses of the AS accelerator. Sample with a fixed *w*/*c* of 0.45 contained 0%, 4%, and 9% AS accelerator, respectively; (**b**) samples with different *w*/*c*. Sample with a fixed AS accelerator of 9% contained 0.40, 0.45, and 0.50 *w*/*c*, respectively.

The hydration heat flow and energy-released curves of samples (PA0-450, PA4-450, PA9-450) were tested with the isothermal calorimeter, and the results are presented in Figure 5a.

During the pre-induction period, there was an initial exothermic peak of 14.7 mW/g for PA0-450. Early hydration is dominated by C_3_A and C_3_S reactions, as described in Equations (1) and (2). The induction period lasted for 7.2 h under low heat evolution, and when the C-S-H nucleated, the liquid phase with Ca^2+^ became supersaturated, and the solubility of C_3_S decreased modestly. In the second exothermic peak, the power level reached 2.3 mW/g at about 19.6 h due to the hydration of C_3_S [40]. Approximately 22.4 h later, a shoulder on the right side of the second exothermic peak, corresponding to the secondary formation of ettringite, was observed.

(1) $$C_{3}A+C\bar{S}H_{2}\left(Gypsum\right)+26H_{2}\to C_{6}A{\bar{S}}_{3}H_{32}\left(AFt\right)$$

(2) $$C_{3}S+\left(3-x-n\right)H\to C_{x}SH_{n}+\left(3-x\right)CH$$

Simplified notation:$\;\bar{S}:SO_{3};H_{2}$:2$H_{2}O$.

The first exothermic peak of AS-accelerated cement pastes was more intense compared to the unaccelerated one, with peaks of 41.2 and 59.5 mW/g of for PA4-450 and PA9-450, respectively. This was due to the rapidly forming ettringite and dissolving C_3_S caused by the AS accelerator’s acid. Ettringite here was formed as a result of Al_2_(SO_4_)_3_ being supplied by the AS accelerator (see Equation (3)). Afterward, the AS accelerator resulted in a shorter induction period for PA4-450 and PA9-450, breaking the chemical equilibrium of liquid-phase calcium ions [41] and accelerating the dissolution of C_3_S and gypsum. The AS accelerator cement paste samples of exothermic peaks were more intense. The exothermic peaks of PA4-450 and PA9-45 were greater than that of PA0-450, with values of 3.1 and 3.3 mW/g, respectively. It could be because the C_3_A reaction occurred earlier, and the heat released by it overlapped with the peak of the C_3_S reaction. As is shown in Figure 5a, the C_3_A reaction peak of PA4-450 almost overlapped with the silicate reaction peak, and the C_3_A reaction peak of PA9-450 appeared on the left side of the silicate reaction peak, showing that the AS accelerator could promote the C_3_A reaction. It is that the low gypsum content of cement accelerates the C_3_A reaction [42,43], which is the high final C_3_A/SO_3_ ratio [31]. Considering that the AS accelerator reacts rapidly with gypsum to form ettringite, cement paste samples with different doses of the accelerator will have a different final C_3_A/SO_3_ ratio. The final C_3_A/SO_3_ ratios for PA0-450, PA4-450, and PA9-450 were 0.48, 0.56, and 0.70, respectively, indicating that cement with more accelerator yields a higher final C_3_A/SO_3_ ratio and a faster C_3_A reaction.

(3) $$6CH+2{\mathrm{Al}}^{3+}+3{\mathrm{SO}}_{4}^{2-}+26H_{2}\to C_{6}A{\bar{S}}_{3}H_{32}\left(AFt\right)$$

According to these results, the AS accelerator affected the hydration kinetics in the early stages of hydration by shortening the hydration induction period of cement and significantly accelerating C_3_A hydration. The energy release of PA0-450, PA4-450, and PA9-450 at 12 h was 28.7 J/g, 82.8 J/g, and 129.4 J/g, respectively. However, the difference in energy release between cement samples with and without the AS accelerator decreased during 12~36 h. The accelerated samples released 55.2~71.3 J/g more energy at 24 h than the others, and 19.1~27.4 J/g more at 36 h. The longer the time, the closer the amounts released are to each other generally. This indicates that the AS accelerator promotes the early hydration reaction of cement and accelerates the release of heat, but its effects are mainly limited to the early stages of hydration.

The hydration heat flow and energy-released curves of cement paste samples with different *w*/*c* were tested with the isothermal calorimeter, and the results are presented in Figure 5b. Cement paste samples with different *w*/*c* exhibited similar heat flow curves. However, cement paste with a higher *w*/*c* displayed a slightly longer induction period, a slightly delayed second exothermic peak, and higher energy released at 36 h. Therefore, it concludes that the change in the *w*/*c* does not have a significant effect on cement hydration kinetics.

### 3.2. Hydrate Assemblage

**Figure 6:**
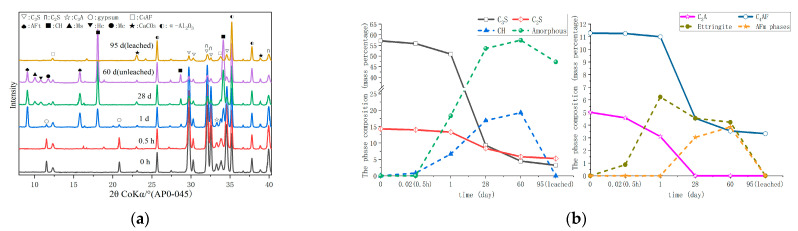
The phase composition of hydrated and leached PA0-045 cement pastes: (**a**) XRD; (**b**) the content of phase composition.

In Figure 6, XRD test results and phase composition of PA0-045 of various ages are presented. Figure 6b was obtained by the Rietveld analysis based on Figure 6a, which had a detailed description in Section 2.2.1. At 0.5 h, a weak ettringite diffraction peak signal was observed when the hydration reactions progressed slowly. The content of C_3_S and C_3_A decreased, as described by Equations (1) and (2), respectively. After the induction period, C_3_S reacted in large quantities during 11.5 h and 24 h in Figure 5a, resulting in the formation of many calcium hydroxides and C-S-H gels at 1 d. The content of C_3_A and gypsum both decreased at that point, while ettringite content increased. As compared to C_3_S, C_2_S is significantly less reactive, while C_4_AF is significantly less reactive than C_3_A. In addition, there were a few minor changes made to the content of C_2_S and C_4_AF. As the C_3_S and C_2_S hydration reaction, more calcium hydroxide and C-S-H gel formation were observed at 28 d. The gypsum was consumed, and the C_3_A and C_4_AF successively reacted with ettringite to produce monosulfate (Ms), as described in Equations (4) and (5) [44,45]. Monosulfate (Ms) [46] forms Hemicarbonate (Hc) and Monocarbonate (Mc) with calcium carbonate. AFm phases [27] include Ms, Mc, and Hc. From 28 d to 60 d, the C_3_S and C_2_S were consumed at a slower rate.

(4) $$2C_{3}A+C_{6}A{\bar{S}}_{3}H_{32}\left(AFt\right)\to 3C_{4}A\bar{S}H_{12}\left(Ms\right)$$

(5) $$2C_{4}AF+C_{6}A{\bar{S}}_{3}H_{32}\left(AFt\right)+12H\to 3C_{4}A\bar{S}H_{12}\left(Ms\right)+2FH_{3}+2CH$$

**Figure 7:**
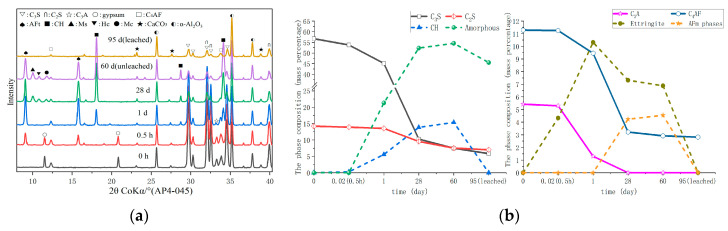
The phase composition of hydrated and leached PA4-045 cement pastes: (**a**) XRD; (**b**) the content of phase composition.

**Figure 8:**
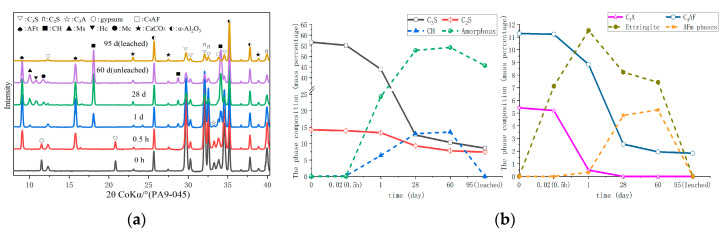
The phase composition of hydrated and leached PA9-045 cement pastes: (**a**) XRD; (**b**) the content of phase composition.

The PA4-045 and PA9-045 of XRD test results and phase composition at various ages are presented in Figure 7 and Figure 8, respectively. Figure 7b and Figure 8b were obtained by the Rietveld analysis based on Figure 7a and Figure 8b, respectively, which had a detailed description in Section 2.2.1. In comparison to PA0-045, PA4-045 and PA9-045 produced more ettringite at 0.5 h, whereas gypsum was consumed more as a result of the AS accelerator [30]. Observation of calcium hydroxide is difficult due to the absence of Ca^2+^ in the AS accelerator, which is required to produce ettringite [47]. The AS accelerator shortened the hydration induction period of cement, and the hydration reaction was accelerated. The consumption of C_3_S in PA4-045 and PA9-045 was, respectively, 93.5% and 111.0% higher than that in PA0-045 at 1 d. Figure 7 and Figure 8 demonstrated that PA4-045 and PA9-045 consumed more C_3_S than PA0-045 at 1d. The gypsum of the AS accelerator cement pastes (PA4-045 and PA9-045) had been consumed, and the consumption of C_3_A and C_4_AF at this time was greater than the cement paste without accelerator (PA0-045), indicating that the AS accelerator promoted the aluminate reaction. The consumption of C_4_AF was low during this period, so the aluminate reaction peak in the heat flow curves was mainly released by the C_3_A reaction. The C_3_S, C_2_S, and C_4_AF underwent hydration reactions from 1 d to 28 d. Due to the low level of C_3_A in the AS accelerator cement samples at 1d, the hydration reaction of C_4_AF was accelerated, resulting in more AFm phase formations at 28 d, as described in Equation (5). Additionally, the C_3_S and C_2_S content of PA4-045 and PA9-045 was slightly higher than that of PA0-045. The results indicated that cement containing AS accelerator inhibited the silicate reaction at 28d. The hydration space for C_3_S and C_2_S was limited due to a large amount of AFt and AFm phases [25,26,48] formed at the early stage in the AS accelerator cement. The later silicate reaction would be adversely affected by this effect. For PA4-045 and PA9-045 than that of PA0-045, the consumption of C3S was 38.1% and 53.4%, respectively lower; the consumption of C_2_S was 20.2% and 36.8% lower at 60 d. Accordingly, The C_3_S content in PA0-045 is 4.5% at 60 d, which is less than that of PA4-045 (7.5%) and PA9-045 (10.3%). Additionally, the content of C_2_S for PA0-045, PA9-045, and PA9-045 were 5.8%, 7.5%, and 7.8%, respectively, at 60 d. The results demonstrate that the AS accelerator has a negative effect on the late hydration process and that this process will be aggravated by higher doses. Calcium hydroxide and C-S-H gel are mainly formed as a result of the silicate reaction. As a consequence, the calcium hydroxide content of PA4-045 and PA9-045 was less than 20.4% and 29.7% than that of PA0-045, and the C-S-H gel content was less than 5.1% and 8.2% than that of PA0-045 at 60 d. In addition, calcium hydroxide and sodium sulfate gel have a considerable effect on the alkalinity and porosity of cement, which ultimately influence its degradation.

In the leached samples, the changed phases were mainly calcium hydroxide, ettringite, AFm phases, and C-S-H gel. Due to the lack of visible peaks, calcium hydroxide, ettringite, and AFm phases were considered completely dissolved. Compared with unleached samples, the approximate content of C-S-H gel in the leached sample decreased by 13.2~17.7% by analyzing the content of Amorphous. The C-S-H gel is partially dissolved and becomes a loose porous structure [22].

### 3.3. Microstructure

#### 3.3.1. SEM

**Figure 9:**
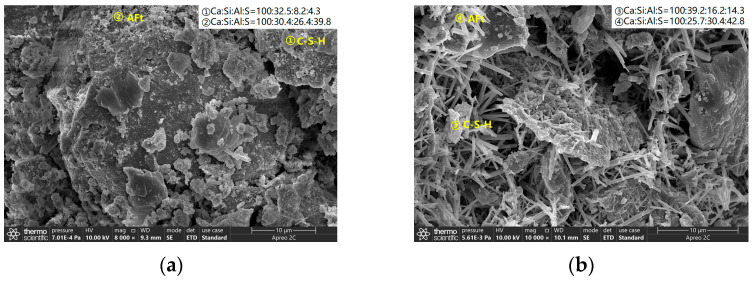
The microstructure of cement samples at 0.5 h: (**a**) PA0-045-0.5h; (**b**) PA9-045-0.5h.

The microstructure of PA0-045 and PA9-045 at 0.5 h are shown in Figure 9. Without an accelerator, cement hydration was relatively slow at the beginning. Consequently, a small number of hydration deposits were observed on the surface of the cement particles in Figure 9a. Among these precipitates were primarily C-S-H gels (Ca/Si equal to 1.91) and AFt (Al/S equal to 0.66). Due to the action of the accelerator, a large number of slender rod-shaped AFt (Al/S equal to 0.71) were observed around the cement particles in the PA9-045 at 0.5 h. These ettringites with slender rod-shaped shapes overlapped each other to form a spatial network structure that filled a large number of cement pores and speeded up cement settings [49].

**Figure 10:**
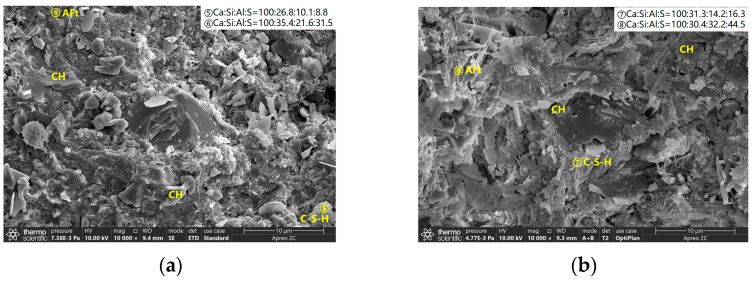
The microstructure of cement samples at 60 d: (**a**) PA0-045-60d; (**b**) PA9-045-60d.

The microstructure of PA0-045 and PA9-045 at 60 d are shown in Figure 10. Several hydration products, including calcium hydroxide (CH), C-S-H gel, and ettringite (AFt), were observed on the surface of the PA0-045 sample. PA9-045 contained similar hydration products, more ettringites, and less calcium hydroxide, as indicated by the results of the XRD analysis. Since PA9-045 had more microscopic pores that were mainly distributed on the disordered overlapping positions of ettringites, it is assumed that PA0-045 possesses a denser microstructure.

**Figure 11:**
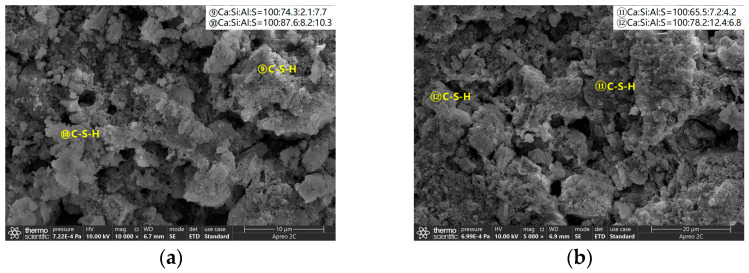
The microstructure of leached cement samples: (**a**) PA0-045-leached; (**b**) PA9-045-leached.

After leaching, a large number of pores appeared on the surface of the samples in Figure 11. Calcium hydroxide and ettringite disappeared, which were in general agreement with the XRD test results. The C-S-H gel was partially dissolved [50,51,52], presenting a loose porous microstructure and a decrease in Ca/Si due to Ca-leaching [6,22], as shown in Figure 11. Compared to the sound samples, the leached present a loose porous microstructure, resulting in a reduction in strength [53]. 

#### 3.3.2. Pore Structure

1. The pore structure in the hydration stage

**Figure 12:**
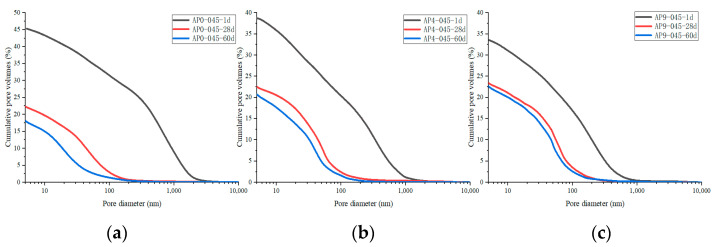
The porosity of hydrated cement pastes with different dosages of the AS accelerator: (**a**) PA0-045; (**b**) PA4-045; (**c**) PA9-045.

For comparison, the pore volume in different cement pastes was normalized to the porosity. Figure 12 shows the porosity of the cement samples with different doses of AS accelerator (PA0-045, PA4-045, and PA9-045). The porosity of all cement samples decreased with curing age due to continuous cement hydration. The AS-accelerated samples at early ages had a lower porosity than the unaccelerated samples, which was reversed at late ages. As early hydration reaction was promoted by the AS accelerator, the porosity of the PA4-045 and PA9-045 were, respectively, 38.7% and 33.2%, which were lower than that of PA4-045 (45.1%). The porosity of the PA0-045, PA4-045, and PA9-045 was 22.3%, 22.5%, and 23.4%, respectively, at 28 d. The porosity of the AS accelerator cement pasts decreased less than that of the unaccelerated cement pasts from 1 d to 28 d due to the adverse impact of the AS accelerator. As the silicate reaction of the AP0-045 sample was more complete at 60 d, the porosity of the AP0-045 sample was lower than that of the PA4-045 and PA9-045 samples. It is proved, therefore, that the higher the dose of AS accelerator, the higher the porosity of the cement at a later stage. 

**Figure 13:**
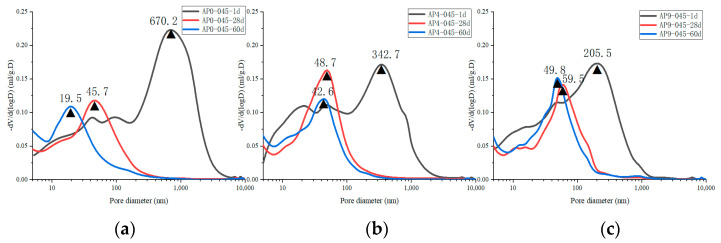
The pore size distribution of hydrated cement pastes with different dosages of the AS accelerator: (**a**) PA0-045; (**b**) PA4-045; (**c**) PA9-045.

Figure 13 shows the pore size distribution of samples with different doses of AS accelerator (PA0-045, PA4-045, and PA9-045). The critical pore diameter [54] is the pore diameter of the largest number of pores in the concrete. The sizes of the pores are divided into four ranges, namely, <10 nm, 10~50 nm, 50~100 nm, and 100~10,000 nm, which correspond to gel-pores, fine-capillary pores, medium-capillary pores, and macro-capillary pores [38,55], respectively. 

**Figure 14:**
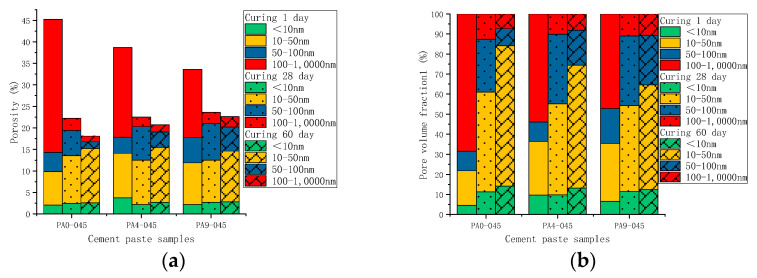
The pore distribution of hydrated cement pastes with different dosages of the AS accelerator: (**a**) the porosity; (**b**) the pore volume fraction.

A change in the pore size distribution within the cement samples also occurred as a result of the hydration reaction. The hydration reaction in the early stages was relatively limited, and the macro-capillary pores in the sample pores had a high porosity and pore volume fraction. The cement samples mixed with the AS accelerator massively formed ettringite which then filled the pores to greatly reduce the porosity of the macro-capillary pores. Compared with the non-accelerator cement sample (PA0-045), the porosity of the macro-capillary pores of the AS accelerator cement samples (PA4-045 and PA9-045) decreased by 32.6% and 48.7% at 1d, respectively. Additionally, the pore volume fractions of the macro-capillary pores of cement with the AS accelerator decreased by 21.2% and 30.1% in Figure 14b, respectively, resulting in a larger critical pore size for unaccelerated cement paste. 

With the increase in curing age, the porosity and pore volume fraction in the samples changed. From day 1 d to day 28 d, the porosity and the pore volume fraction of macro-capillary pores dropped significantly, reducing the critical pore size at 28 d from 205.5~670.2 nm to 45.7~59.5 nm. AP0-045 went through the most remarkable change; its critical pore size fell from 670.2 nm to 45.7 nm. In response to the late silicate reaction, the pore volume fraction of gel pores increased, and that of fine-capillary pores and medium-capillary pores decreased in Figure 14b. Thus, the critical pore diameter of the cement sample was reduced to 19.5~49.8 nm at 60 d, and the critical pore diameter of PA0-045, PA4-045, and PA9-045 was 19.5 nm, 42.6 nm, and 49.8 nm, respectively. Due to the effect of the AS accelerator on the late silicate reaction, the accelerated cement pastes contained less C-S-H gel (to be shown in Figure 7b and Figure 8b), and the pore volume fraction of the gel pores was lower, resulting in a larger critical pore size. The pore structure of the accelerated sample was significantly less dense than that of the unaccelerated cement sample at the later stages of hydration, which was in accord with the results of the SEM microscopy (to be shown in Figure 10). In conclusion, the AS accelerator is beneficial to the pore structure of cement at the early stage but then negatively affects the pore size at the later stages. 

**Figure 15:**
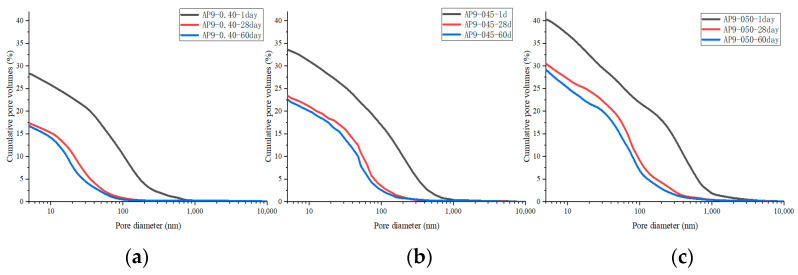
The porosity of hydrated AS-accelerated cement pastes with different *w*/*c*: (**a**) PA9-040; (**b**) PA9-045; (**c**) PA9-050.

**Figure 16:**
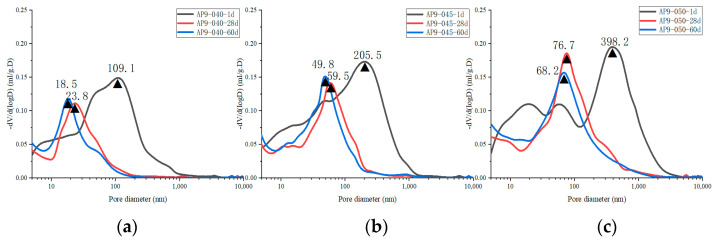
The pore size distribution of hydrated AS-accelerated cement pastes with different *w*/*c*: (**a**) PA9-040; (**b**) PA9-045; (**c**) PA9-050.

**Figure 17:**
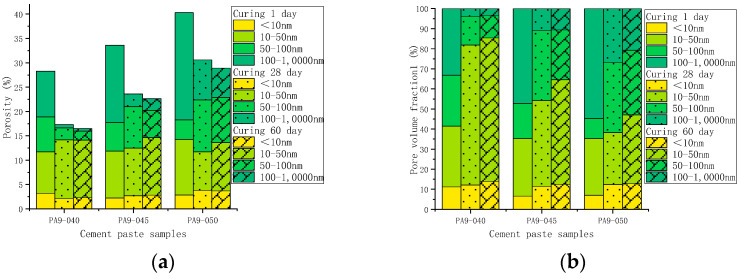
The pore distribution of hydrated cement pastes with different *w*/*c*: (**a**) the porosity; (**b**) the pore volume fraction.

Figure 15 shows the porosity of AS accelerator cement with different *w*/*c*. The porosity of high *w*/*c* cement pastes with the AS accelerator was lower at various curing ages. The results of the heat flow curves indicated that *w*/*c* did not significantly affect the hydration kinetics of cement with the AS accelerator. However, in cement with a high *w*/*c*, there were fewer cementitious materials in the monomer volume cement, which resulted in a higher porosity after evaporation of water. As is shown in Figure 16 and Figure 17, samples with high *w*/*c* had a higher pore volume fraction of the macro-capillary, resulting in a larger critical pore size. The higher the *w*/*c*, the looser the AS cement pore structure, a rule similarly found in the pore structure of ordinary cement.

2. The pore structure before and after leaching

**Figure 18:**
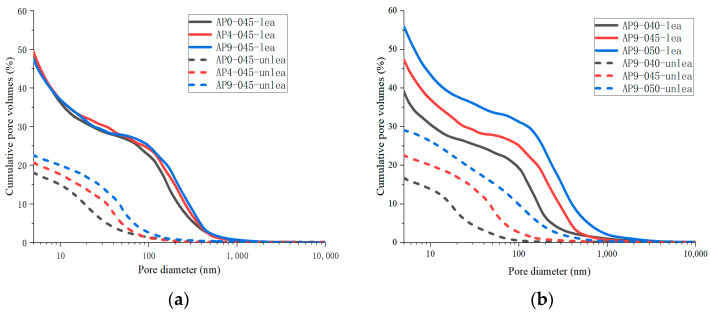
The porosity of the unleached and leached cement pastes: (**a**) different dosages of the AS accelerator; (**b**) different *w*/*c*.

Figure 18 illustrates the changes in the porosity of all cement samples before and after leaching. There was a significant increase in porosity in all test cases after leaching. Compared with unleached ones, the porosity of leached samples increased by 93.1~170.1%. A direct relationship between the porosity of the leached samples and the *w*/*c* was also observed in Figure 18. The higher the *w*/*c*, the higher the porosity of the leached samples. The porosity of the leached samples was similar despite the different doses of accelerator in the samples (PA0-045, PA4-045, and PA9-045).

**Figure 19:**
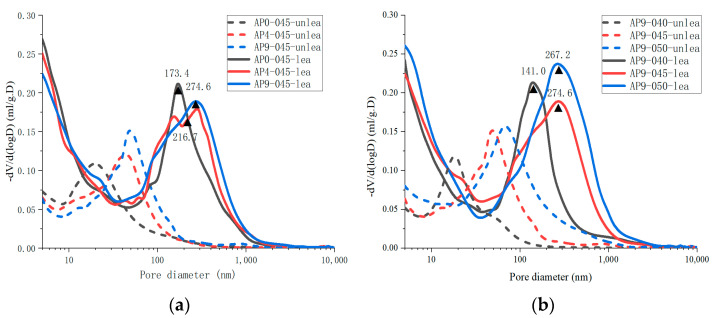
The pore size distribution of the unleached and leached cement pastes: (**a**) different dosages of the AS accelerator; (**b**) different *w*/*c*.

**Figure 20:**
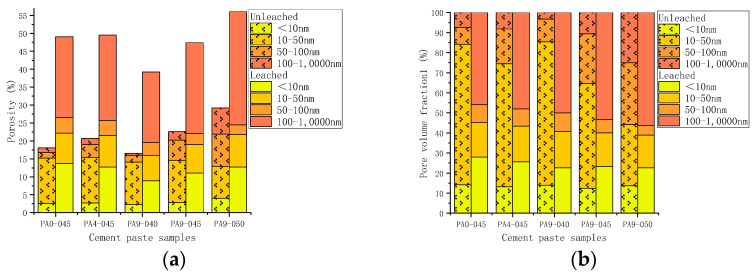
The pore distribution of the unleached and leached cement pastes: (**a**) the porosity; (**b**) the pore volume fraction.

Figure 19 illustrates the changes in pore size distribution of all cement samples before and after leaching.Figure 20 shows that the porosity and pore volume fraction of fine-capillary pores and medium-capillary pores decreased after leaching, whereas gel pores and macro-capillary pores showed an increase. The XRD analysis revealed that the hydration products dissolved in all cement pastes were mainly calcium hydroxide, aluminate hydrate, and C-S-H gel. Pore sizes for calcium hydroxide [6] and aluminate hydrate [56] were 60~500 nm and 10~100 nm, respectively. The dissolution of calcium hydroxide and aluminate hydrate explains the decrease in the porosity and pore volume fraction of fine-capillary and medium-capillary pores. C-S-H gels [57] had a maximum pore size of 20 nm and were partially dissolved, which led to an increase in gel pores. The range of critical pore diameter of all samples after leaching was mostly 141.0~274.6 nm, while that before leaching was 18.5~68.2 nm in Figure 19.

### 3.4. The Leaching Length

**Figure 21:**
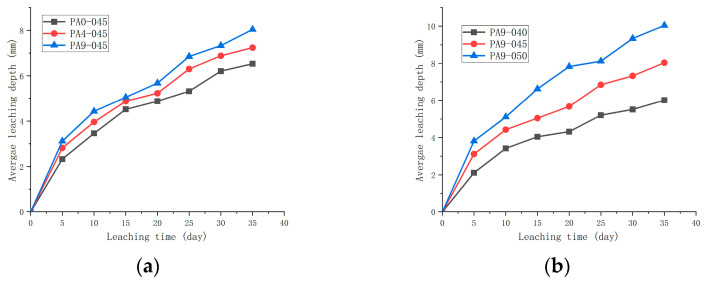
Changes in the average leaching depth of samples in 6M NH_4_Cl solution at 1cm/s with leaching time: (**a**) different dosages of the AS accelerator; (**b**) different *w*/*c*.

Figure 21 shows the average leaching depth, measured by phenolphthalein, of the samples. The increase in the average leaching depth was initially rapid but gradually slowed down with leaching time. As is shown in Figure 21, both the dose of the AS accelerator and the *w*/*c* directly influenced the average leaching depth. The average leaching depths of samples (PA0-045, PA4-045, and PA9-045) increased with doses of the AS accelerator, showing that the AS accelerator accelerated Ca-leaching. In addition, the average leaching depths of samples (PA9-040, PA9-045, and PA9-050) increased with *w*/*c* in the AS accelerator cement samples.

**Figure 22:**
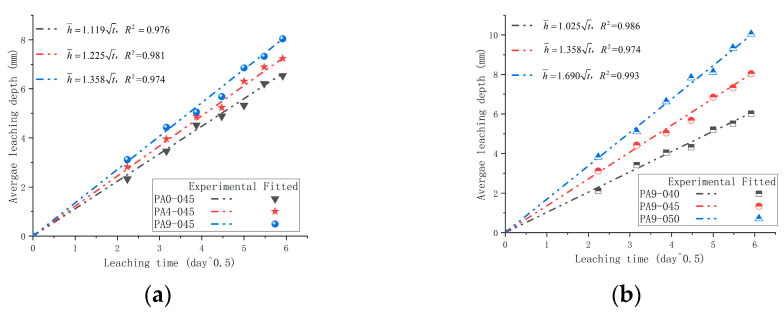
Relationship between the average leaching depth$\;\bar{h}$ and $\sqrt{t}$ for samples in 6M NH_4_Cl solution at 1 cm/s: (**a**) different dosages of the AS accelerator; (**b**) different *w*/*c*.

To further determine the relationship between the average leaching depth and leaching time, the test data from Figure 21 were numerically fitted to the formula $\bar{h}=k\sqrt{t}$ (where $\bar{h}$ = leaching depth; k = leaching constant; and t = leaching time). According to Berra et al. [58], the leaching constant (k) can be used as an indicator of leaching resistance. Additionally, k is a parameter that relates to the AS accelerator dosage and *w*/*c* in this paper. The leaching constants (k) of cement with different doses of accelerators (PA0-045, PA4-045, and PA9-045) were 1.119, 1.225, and 1.358, respectively, in Figure 22. Thus, an AS accelerator reduces cement’s leaching resistance, and a higher dosage of it has a more pronounced effect. According to Figure 12, samples with high leaching constants have a looser pore structure, making them more susceptible to leaching. The leaching constants k of AS accelerator cement with different *w*/*c* (PA9-040, PA9-045, and PA9-050) were 1.025, 1.358, and 1.690, respectively, in Figure 22. As a result of the looser pore structure of samples with a higher leaching constant, the samples are more prone to leach. Therefore, reducing the *w*/*c* of AS accelerator cement pastes will improve its leaching resistance. 

## 4. Discussion

Based on the heat flow analysis and the hydrate assemblage, it can be concluded that the AS accelerator affects the early hydration reaction by producing more ettringite, resulting in a shorter induction period of cement. As a result of the AS accelerator promoting cement hydration, samples containing the AS accelerator release more energy than those without it on the first day. Silicate hydration is promoted in AS accelerator samples. The consumption of C_3_S in PA4-045 and PA9-045 were, respectively, 93.5% and 111.0% more than that of PA0-045 at 1 d. As a result, cement paste containing AS accelerator has a lower porosity and a smaller critical pore size in its early stages. Furthermore, a higher dose of AS accelerator has a denser pore structure. However, the AS accelerator hinders the late silicate hydration. When the Al^3+^/SO_4_^2−^ of the AS accelerator exceeds 0.33 [31], the consumption of sulfate in the cement is accelerated, causing the C_3_A reaction to occur before the main silicate hydration in Figure 5a and inhibiting late silicate hydration. The accelerated samples released 55.2~71.3 J/g more energy at 24 h than those without it, and 19.1~27.4 J/g more at 36 h. Additionally, the AS accelerator samples’ hydration C_4_AF reaction is accelerated, resulting in more AFm phase formations in the later stage. The total consumption of C_3_S and C_2_S in PA4-045 and PA9-045 were, respectively, 7.7% and 12.8% less than that of PA0-045 at 60 d, resulting in an accelerated cement with higher porosity and rougher pore structure (to be shown in Figure 12 and Figure 13). A higher dose of AS accelerator has a rougher pore structure in the later stage.

Cement with a loose pore structure is less resistant to leaching [22]. This aligns with the results of the RWN test, in which the AS accelerator cement samples had a significantly deeper average leaching depth at the same leaching time. In comparison with the non-accelerated cement sample with *w*/*c* = 0.45, the leaching constants (k) of AS-accelerated cement increased by 9.5–21.3%, respectively, when the AS accelerator dosage was 4–9%. In other words, the leaching resistance of cement will reduce by 9.5–21.3%. The AS accelerator reduces the leaching resistance of shotcrete, an interaction detrimental to the long-term safety of tunnels in rich water mountainous regions. The reduction in the *w*/*c* did not have a significant effect on the cement’s hydration kinetics, but it could improve the cement’s pore structure, which ultimately contributed to leaching resistance. Based on the cement with a *w*/*c* of 0.45 and an AS accelerator content of 9%, reducing the *w*/*c* of the cement from 0.45 to 0.40 results in a 24.5% improvement in leaching resistance.

Leaching causes calcium hydroxide, ettringite, and AFm phases to completely dissolve, and C-S-H gel to partially dissolve, altering cement microstructure. The porosity of the cement samples increased substantially, as is observed in leached samples which increased in porosity from 93.1% to 170.1% compared with unleached ones. In the leached samples, the porosity and pore volume fraction of small and medium capillary pores decreased significantly, while that of large capillary pores and gel pores increased. As a result, the critical pore size of the leached samples increases from 18.5~68.2 nm to 141.0~274.6 nm. As a result of Ca-leaching, cement pastes form rough pore structures, which reduce their strength and pose a threat to tunnel structures.

This paper examines the adverse effects of the AS accelerator on cement Ca-leaching resistance, taking a common dosage of the AS accelerator used in shotcrete in tunnel engineering as an example. This indicates that the tunnel shotcrete is experiencing a more serious Ca-leaching problem. Based on the findings of this paper, the Railway Operating Company can adjust the operation and maintenance of the tunnels in water-rich mountains and guide the design of the tunnels being planned.

There are other ways to improve the leaching resistance of concrete besides reducing *w*/*c*. There has been evidence that the substitution of clinker with supplementary cementitious materials (SCMs) such as limestone, slag, and silica fume dramatically improves the pore structure and leaching resistance of cement without accelerators, but research on this subject has not yet been conducted in cement with accelerators. Combining artificial intelligence technology with machine learning to develop an alternative model [59] for the study of the AS accelerator cement with leaching with varied parameters (such as cement content and SCMs content) will save considerable research time and serve as a hot research direction for the future. Furthermore, the dynamic response [60] of the reciprocating train load [61] to the bottom shotcrete structure with leaching is a serious issue, and corresponding research will be conducted in the future. 

## 5. Conclusions

The purpose of this paper is to examine the effects of the AS accelerator and *w*/*c* on cement paste hydration and leaching, and the following conclusions can be drawn from the above test results:

(1) The AS accelerator changes the pore structure of cement by altering its hydration kinetics. A cement paste containing a higher dose of the AS accelerator has a lower porosity and a smaller critical pore size in the early stage, and a higher porosity and a larger critical pore size in the later stage;
(2) The hydration kinetics of AS cement pastes are not significantly affected by *w*/*c*. As the *w*/*c* decreases, the porosity decreases, and the critical pore size of the cement with the AS accelerator decreases;
(3) Leaching changes the microstructure of cement. Compared with unleached ones, the porosity of leached samples increased by 93.1–170.1%. The higher the *w*/*c*, the higher the porosity of the leached samples. Despite samples containing different doses of the AS accelerator, the porosity of the leached samples with the same *w*/*c* is slightly similar;
(4) It is clear that the AS accelerator affects the leaching resistance of shotcrete. As compared with unaccelerated cement samples, cement leaching resistance decreases by 9.5~21.3% when the AS accelerator dosage is 4~9%;
(5) The reduction in the *w*/*c* contributes to leaching resistance. The reduction in *w*/*c* from 0.45 to 0.40 results in a 24.5% improvement in leaching resistance based on cement with a *w*/*c* of 0.45 and an AS accelerator content of 9%.

## Figures and Tables

**Table 1:** Chemical composition of Portland cement.

Composition	CaO	SiO_2_	Al_2_O_3_	Fe_2_O_3_	Na_2_O	MgO	K_2_O	Ignition Loss
Content (wt%)	63.21	20.35	4.52	3.75	0.06	1.78	0.74	1.67

**Table 2:** The mineralogical phase composition of Portland cement.

Clinker Mineral	C_3_S	C_2_S	C_3_A	C_4_AF
Content (wt%)	57.11	14.32	5.02	11.28

**Table 3:** The basic composition of the aluminum sulfate-based alkali-free accelerator (liquid).

Composition	Al_2_O_3_	SO_4_^2−^	Na_2_O	pH (20 ℃)	Solid Content	Density (g/cm^3^)
Content (wt%)	13.6	25.6	-	2.8	52.5	1.39

**Table 4:** Cement paste compositions after adding the AS accelerator.

Name		AP0-045	AP4-045	AP9-045	AP9-040	AP9-050
Cement	(g)	100	100	100	100	100
Deionized water	(g)	45	45	45	40	50
AS accelerator	(% bcw)	0	4	9	9	9

Note: the meaning of “% bcw” is “by cement weight“.

## Data Availability

The data that support the findings of this study are available on request from the corresponding author, D.L., upon reasonable request.
